# Combined Effects of Seed-Inoculated PGPR and Foliar-Applied Pistachio Hull Wood Vinegar on Salinity Stress Alleviation in Broad Bean (*Vicia faba* L.)

**DOI:** 10.3390/plants15213360

**Published:** 2026-10-29

**Authors:** Özge Uçar

**Affiliations:** Department of Field Crops, Faculty of Agriculture, Siirt University, 56100 Siirt, Türkiye; ozge.ucar@siirt.edu.tr

**Keywords:** broad bean, PGPR bacteria, MDA, EL, salinity, wood vinegar, pistachio hull, K^+^/Na^+^

## Abstract

One of the main abiotic factors that restricts plant growth is salinity, which has a negative impact on a variety of physiological processes. One environmentally friendly method used to lessen the detrimental effects of salt stressors on plants is the application of native stress-tolerant plant growth-promoting rhizobacteria (PGPR) with combined foliar application of wood vinegar (WV). This study aims to evaluate the effectiveness of seed inoculation with the plant growth-promoting rhizobacterium (PGPR) *Paenibacillus xylanilyticus* strain KF63C, in combination with the foliar application of wood vinegar (WV) derived from regional Siirt pistachio fruit hull biomass, in mitigating the adverse effects of salinity stress (150 mM NaCl) in broad bean (*Vicia faba* L. cv. Salkım). A 30-day pot experiment was conducted under controlled growth chamber conditions to investigate the effects of two salinity levels (0 and 150 mM NaCl) in combination with six protective treatments: control, PGPR, 0.50% WV, 1.00% WV, PGPR + 0.50% WV, and PGPR + 1.00% WV. The results revealed that high salinity stress (150 mM NaCl) significantly restricted vegetative growth, degraded photosynthetic pigments, and “altered ionic balance, particularly K^+^ and Na^+^ accumulation” while increased electrolyte leakage (EL) and malondialdehyde (MDA) accumulation, indicating enhanced membrane damage and lipid peroxidation. Although PGPR inoculation alone improved shoot elongation, its effects on the other physiological traits were variable. Among the tested treatments, PGPR + 0.50% WV showed the strongest numerical response. This combined treatment distinctly minimized oxidative and membrane damage (yielding the lowest EL and MDA levels), maintained cellular ion balance (maintained ionic balance, as indicated by higher K^+^ accumulation and K^+^/Na^+^ ratios under salinity), and helped to maintain chlorophyll and carotenoid concentrations. In contrast, 1.00% WV showed adverse effects on several growth and physiological traits, indicating the importance of dose optimization. These findings suggest that the combined application of PGPR and 0.50% pistachio hull-derived WV may represent a promising sustainable approach for mitigating salinity-induced damage in faba bean.

## 1. Introduction

Broad bean (*Vicia faba* L., *Fabaceae*) is an important food legume with considerable value for both human nutrition and sustainable crop production. Its grains provide substantial amounts of protein content while its symbiotic association with nitrogen-fixing microorganisms contributes to biological nitrogen fixation and improved soil fertility [1]. However, climate change and abiotic stressors particularly soil salinity severely constrain global broad bean production [2]. Approximately 20% of global agricultural land and over 33% of irrigated areas are currently threatened by salinity [3]. High salt concentrations induce osmotic stress, ion toxicity (accumulations of Na^+^ and Cl^−^), nutrient imbalances, and the overproduction of reactive oxygen species (ROS) that cause cellular damage via lipid peroxidation [4,5]. Classified as moderately sensitive to salinity, *V. faba* exhibits reduced germination rates, stunted growth, chlorophyll degradation, and dramatic yield losses under high-salinity conditions [6,7]. Developing eco-friendly, sustainable mitigation strategies to reduce salt-induced damage is therefore important for maintaining broad bean productivity in salt-affected soils [8].

Biological approaches have received increasing attention as alternatives or complements to conventional chemical inputs. Plant growth-promoting rhizobacteria (PGPR) are beneficial microorganisms that colonize the rhizosphere, promoting plant growth via both direct and indirect mechanisms [9,10]. Under salt stress, PGPR mitigate adverse effects by synthesizing phytohormones like indole-3-acetic acid (IAA), solubilizing phosphate, lowering stress-induced ethylene via 1-aminocyclopropane-1-carboxylate (ACC) deaminase activity, and stimulating the accumulation of osmoregulators such as proline [11,12]. PGPR may also enhance osmotic adjustment, antioxidant defense, and nutrient acquisition, while promoting root development and improving ion homeostasis. In particular, maintenance of a favorable K^+^/Na^+^ balance is an important component of plant adaptation to salinity [13].

Beyond biological interventions, converting agricultural and industrial biomass residues into value-added bio-amendments is crucial for enhancing agro-ecological sustainability. Thermal conversion and chemical functionalization of organic substances have gained substantial interest in buffering plant–soil abiotic stress and facilitating nutrient bioavailability under arid and calcareous soil conditions [14]. Siirt province, located in Türkiye, is a major production hub for pistachio (*Pistacia vera* L.) globally [15]. The Siirt pistachio (*Pistacia vera* L. cv. Siirt), a certified regional cultivar, is morphologically distinct from other cultivars such as the Antep pistachio (*Pistacia vera* L. cv. Antep). The ‘Siirt’ cultivar features significantly larger, plumper, and rounder fruit structures, with a natural on-tree dehiscence (splitting) rate exceeding 90% [16]. This morphological size and high splitting trait generate substantial amounts of outer hull (mesocarp) per fruit during post-harvest processing, resulting in a high volume of agro-industrial biomass waste [17]. Owing to its rich phenolic profile (particularly anthocyanins and flavonoids, which confer its distinct pinkish hue) and potent antioxidant properties, Siirt pistachio hull biomass holds notable bioactive potential [18].

Thermal degradation of this biomass in an oxygen-limited environment (pyrolysis) yields wood vinegar (WV) (pyroligneous acid) as a co-product a complex liquid containing organic acids (predominantly acetic acid), phenolic compounds, and esters [19]. Foliar application of WV at low concentrations has been shown to stimulate plant growth, trigger antioxidant mechanisms, and improve abiotic stress tolerance [20,21]. The bioactive compounds present in WV derived from pistachio hull biomass may therefore provide an additional means of stimulating plant defense and physiological adjustment under unfavorable conditions [22].

Although the individual physiological contributions of PGPR and WV have been documented, information regarding their synergistic or combined application in broad bean (*Vicia faba* L.) under salt stress remains sparse. In particular, limited information is available on the interaction between seed-applied PGPR and foliar-applied WV produced from locally available Siirt pistachio hull biomass. Understanding whether these treatments act independently or complement each other could provide useful information for developing sustainable approaches to salinity management.

The present study therefore evaluated the individual and combined effects of seed inoculation with PGPR and foliar application of pistachio hull-derived WV on broad bean (*Vicia faba* L.) exposed to salt stress under controlled conditions. Particular attention was given to tissue ion balance, oxidative stress, photosynthetic pigments, and early vegetative growth to determine whether these treatments could improve physiological performance and reduce the adverse effects of salinity.

## 2. Results

The two-way analysis of variance (ANOVA) revealed that the main effects of salinity (S), treatments (T), and their interaction (S × T) were statistically significant at the 1% probability level (*p* < 0.01) across all evaluated morpho-physiological and biochemical parameters (Table 1). Furthermore, high partial eta-squared (η_p_^2^) values for the S × T interaction confirmed that the applied treatments markedly modulated the physiological response of broad bean (*Vicia faba* L.) under salinity stress. The detailed responses of individual growth, membrane integrity, ionic balance, and photosynthetic traits are presented in the following subsections.

### 2.1. Effects of Treatments on Shoot and Root Growth

**Figure 1:**
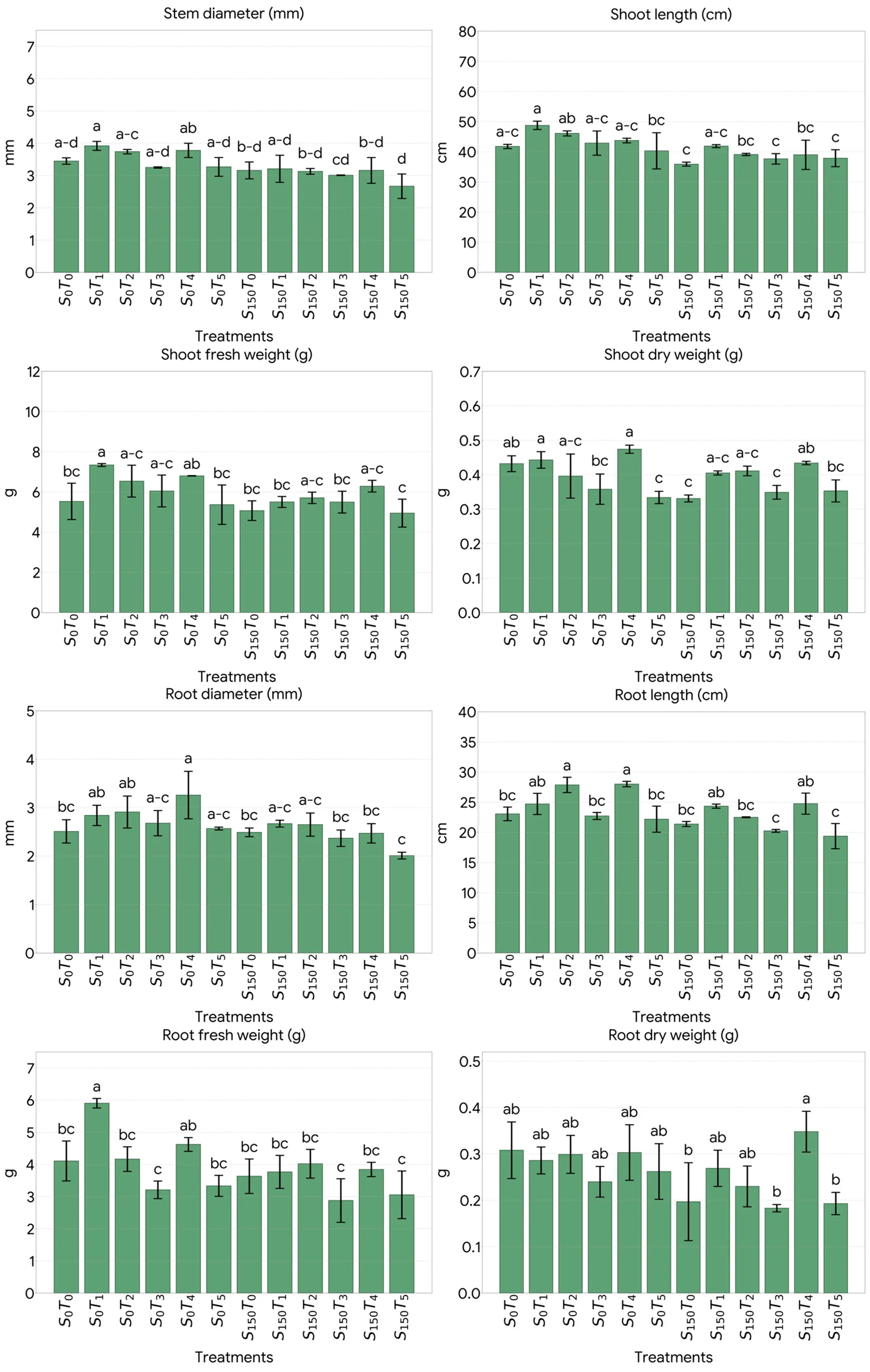
Effects of different treatments on growth parameters of broad bean (*Vicia faba* L.) plants under non-saline (S_0_), and saline (S_150_) conditions. Vertical bars represent mean ± SE of replicates (*n* = 3). Different lowercase letters above the bars indicate statistically significant differences among treatments (*p* < 0.01). S_0_: 0 mM NaCl, S_150_: 150 mM NaCl. T_0_: Control, T_1_: PGPR, T_2_: 0.50% WV, T_3_: 1.00% WV, T_4_: PGPR + 0.50% WV, T_5_: PGPR + 1.00% WV (PGPR: Plant growth-promoting rhizobacteria, *Paenibacillus xylanilyticus* strain KF63C; WV: Wood vinegar, also known as pyroligneous acid).

The effects of the bio stimulant treatments on plant growth parameters are illustrated in Figure 1, with detailed numerical datasets provided in Supplementary Table S1 for shoot characteristics and Supplementary Table S2 for root characteristics. The treatments significantly influenced the shoot and root growth of faba bean plants. Under non-saline conditions (S_0_), the highest values for stem diameter (3.92 mm), shoot length (48.78 cm), shoot fresh weight (7.340 g), and root fresh weight (5.904 g) were recorded in the S_0_T_1_ (bacterial inoculation) group. Conversely, the S_0_T_4_ treatment (bacterial inoculation + 0.50% WV) yielded the highest root diameter (3.26 mm), root length (28.01 cm), and shoot dry weight (0.474 g). Root dry weight also reached its numerically highest value in the S_0_T_4_ treatment (0.303 g), although this value was statistically comparable several other treatments.

Increasing salinity to 150 mM caused marked reductions in plant growth parameters. In control plants subjected to salt stress (S_150_T_0_), shoot length (35.90 cm), shoot fresh weight (5.070 g), root length (21.38 cm), and root dry weight (0.197 g) were lower than those of the S_0_T_0_ group. Under 150 mM NaCl conditions, the highest stem diameter (3.21 mm) and shoot length (41.89 cm) were recorded under S_150_T_1_ and were statistically comparable with S_150_T_4_ (3.16 mm) for stem diameter. In contrast, the S_150_T_4_ treatment exhibited the highest values for shoot fresh weight (6.287 g), shoot dry weight (0.434 g), root length (24.76 cm), and root dry weight (0.348 g). Under 150 mM NaCl conditions, root fresh weight reached its highest numerical value in the S_150_T_2_ treatment (4.020 g), although it remained statistically similar to the S_150_T_1_ and S_150_T_4_ treatments.

The application of 1.0% WV, particularly under salt stress conditions, generally resulted in lower growth values. Under S_150_ conditions, the lowest values for shoot diameter (2.67 mm), root diameter (2.01 mm), root length (19.37 cm), shoot fresh weight (4.944 g), root fresh weight (3.056 g), and root dry weight (0.193 g) were recorded in the S_150_T_5_ group.

### 2.2. Effects of Treatments on Electrolyte Leakage (EL) and Malondialdehyde (MDA) Content

**Figure 2:**
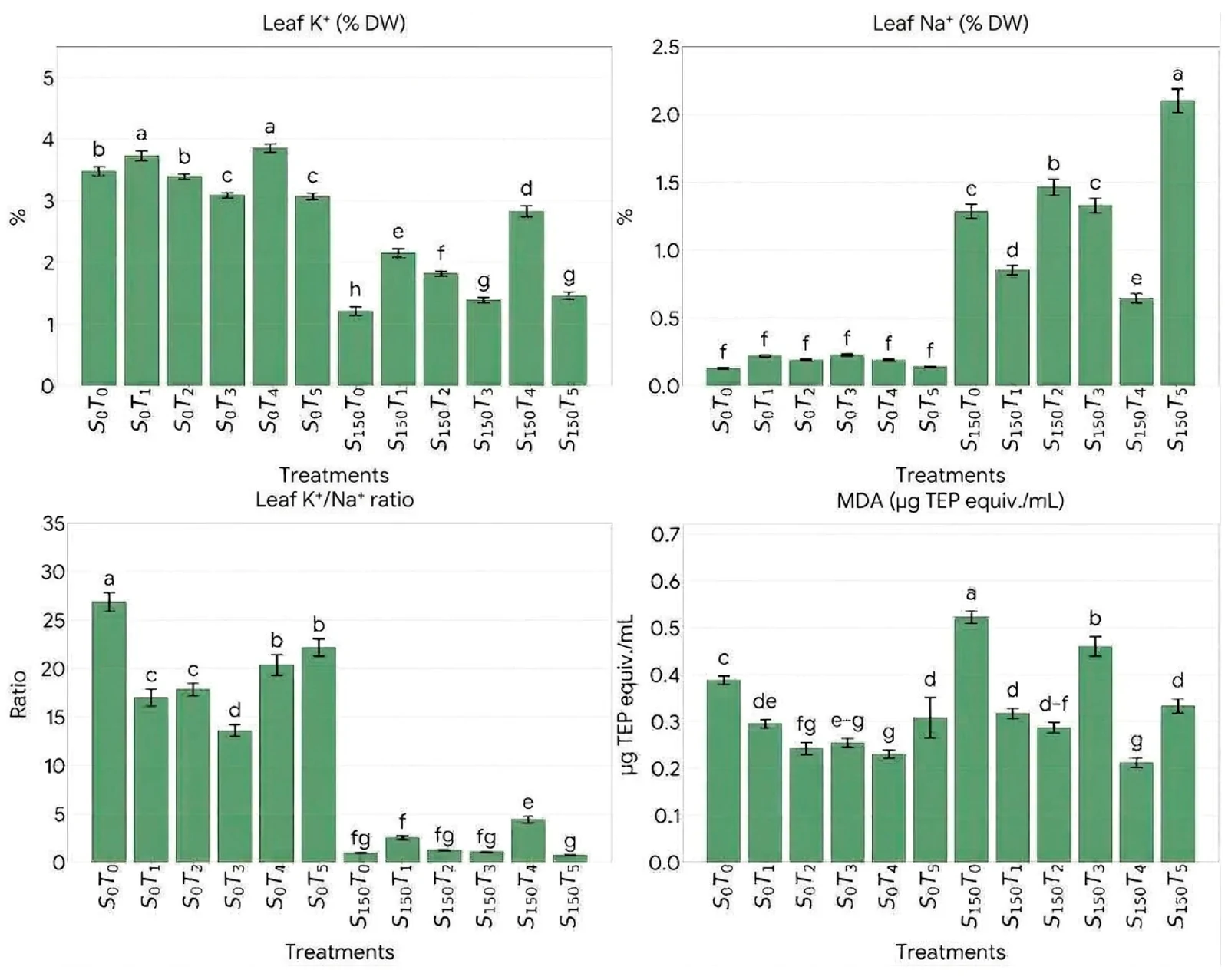
Effects of different treatments on potassium (K^+^), sodium (Na^+^), K^+^/Na^+^ ratio, and malondialdehyde (MDA) content of broad bean (*Vicia faba* L.) plants under non-saline (S_0_), and saline (S_150_) conditions. Vertical bars represent mean ± SE of replicates (*n* = 3). Different lowercase letters above the bars indicate statistically significant differences among treatments (*p* < 0.01). S_0_: 0 mM NaCl, S_150_: 150 mM NaCl. T_0_: Control, T_1_: PGPR, T_2_: 0.50% WV, T_3_: 1.00% WV, T_4_: PGPR + 0.50% WV, T_5_: PGPR + 1.00% WV (PGPR: Plant growth-promoting rhizobacteria, *Paenibacillus xylanilyticus* strain KF63C; WV: Wood vinegar, also known as pyroligneous acid).

**Figure 3:**
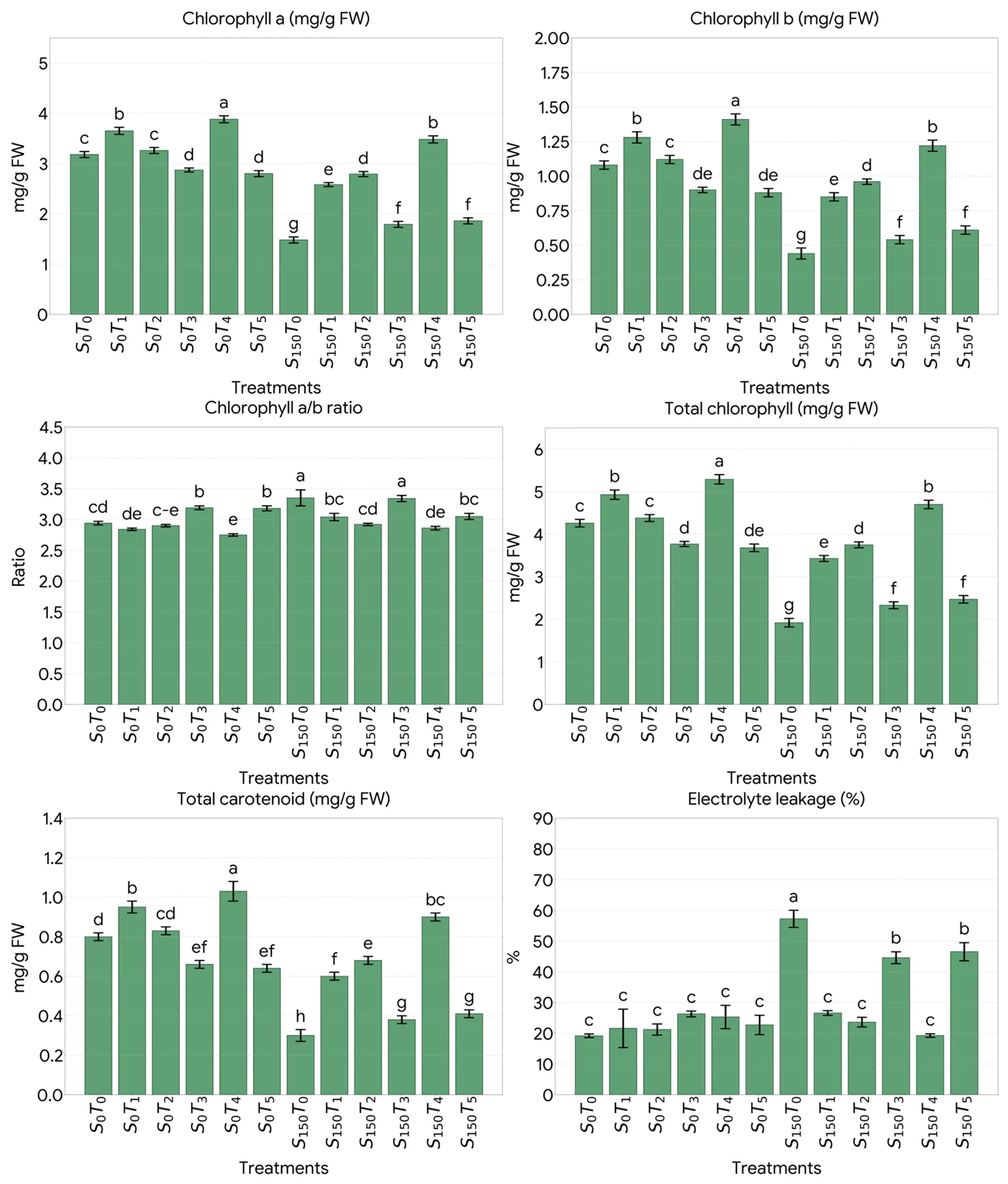
Effects of different treatments on chlorophyll a content, chlorophyll b content, chlorophyll a/b ratio, total chlorophyll content, total carotenoid content, and electrolyte leakage of broad bean (*Vicia faba* L.) plants under non-saline (S_0_), and saline (S_150_) conditions. Vertical bars represent mean ± SE of replicates (*n* = 3). Different lowercase letters above the bars indicate statistically significant differences among treatments (*p* < 0.01). S_0_: 0 mM NaCl, S_150_: 150 mM NaCl. T_0_: Control, T_1_: PGPR, T_2_: 0.50% WV, T_3_: 1.00% WV, T_4_: PGPR + 0.50% WV, T_5_: PGPR + 1.00% WV (PGPR: Plant growth-promoting rhizobacteria, *Paenibacillus xylanilyticus* strain KF63C; WV: Wood vinegar, also known as pyroligneous acid).

The effects of salinity and biostimulant treatments on EL and MDA content are presented in Figure 2 and Figure 3, with corresponding detailed datasets provided in Supplementary Table S3. Salinity stress resulted in a pronounced increase in EL and MDA content. EL rose from 19.19% in the S_0_T_0_ control to 57.24% in the S_150_T_0_ treatment. Similarly, MDA content increased from 0.388 µg TEP equivalent/mL in the S_0_T_0_ group to 0.522 µg TEP equivalent/mL in the S_150_T_0_ group.

Under non-saline conditions, EL ranged between 19.19% and 26.31%, with no statistically significant differences observed among treatments. Conversely, MDA content varied depending on the application; the highest value was recorded in S_0_T_0_ (0.388 µg TEP equivalent/mL), whereas the lowest value was observed in S_0_T_4_ (0.230 µg TEP equivalent/mL). Comparatively low MDA values were also registered in the S_0_T_2_ and S_0_T_3_ treatments.

Under 150 mM NaCl stress, the highest EL (57.24%) and MDA content (0.522 µg TEP equivalent/mL) were reached in the control group (S_150_T_0_). Exogenous applications substantially mitigated these stress indicators. The lowest EL was recorded in S_150_T_4_ (19.26%), followed by S_150_T_2_ (23.62%) and S_150_T_1_ (26.60%). Similarly, the lowest MDA content was determined in the S_150_T_4_ treatment (0.212 µg TEP equivalent/mL), while S_150_T_3_ maintained a relatively high MDA level (0.460 µg TEP equivalent/mL).

### 2.3. Effects of Treatments on K^+^, Na^+^, and K^+^/Na^+^ Ratio

The effects of salinity stress and biostimulant treatments on leaf K^+^, Na^+^, and K^+^/Na^+^ ratio are illustrated in Figure 2, with detailed numerical datasets provided in Supplementary Table S3. Salinity induced significant alterations in the mineral element accumulation of the plants. The K content declined from 3.48% in the S_0_T_0_ application to 1.21% in S_150_T_0_. Conversely, Na content rose sharply from 0.130% to 1.285%. Driven by these shifts, the K^+^/Na^+^ ratio dropped from 26.85 in the S_0_T_0_ group to 0.94 in S_150_T_0_.

In non-saline controls, the highest K^+^ content was recorded in S_0_T_4_ (3.85%), followed by S_0_T_1_ (3.73%). The highest K^+^/Na^+^ ratio was observed in S_0_T_0_ (26.85), whereas the lowest ratio was recorded in S_0_T_3_ (13.59).

Under 150 mM salt stress, the highest K^+^ content (2.83%) and lowest Na^+^ content (0.646%) were recorded in S_150_T_4_ among all salt-stressed applications. The highest K^+^/Na^+^ ratio under stress was maintained in S_150_T_4_ (4.40), followed by S_150_T_1_ (2.53). In contrast, the highest Na^+^ content (2.101%) and the lowest K^+^/Na^+^ ratio (0.70) were registered in the S_150_T_5_ treatment.

### 2.4. Effects of Treatments on Photosynthetic Pigments

The effects of the treatments on the photosynthetic pigment profiles and membrane integrity of broad bean (*Vicia faba* L.) plants are presented in Supplementary Table S4 and illustrated in Figure 3. Chlorophyll and carotenoid contents were significantly influenced by both salinity levels and treatment applications. Under salt stress, the control group (S_150_T_0_) exhibited marked reductions in chlorophyll a, chlorophyll b, total chlorophyll, and total carotenoid contents relative to S_0_T_0_. Chlorophyll a decreased from 3.18 to 1.48 mg/g FW, chlorophyll b from 1.08 to 0.44 mg/g FW, total chlorophyll from 4.26 to 1.92 mg/g FW, and total carotenoid content from 0.80 to 0.30 mg/g FW.

Under non-saline conditions, the highest contents of chlorophyll a (3.88 mg/g FW), chlorophyll b (1.11 mg/g FW), total chlorophyll (5.29 mg/g FW), and total carotenoids (1.03 mg/g FW) were obtained in the S_0_T_4_ application. Pigment accumulation was also remarkably high in S_0_T_1_, with chlorophyll a, chlorophyll b, total chlorophyll, and carotenoid contents reaching 3.65, 1.28, 4.93, and 0.95 mg/g FW, respectively.

Under 150 mM NaCl stress, S_150_T_4_ outperformed all other stress applications, displaying the highest levels of chlorophyll a (3.48 mg/g FW), chlorophyll b (1.22 mg/g FW), and total chlorophyll (4.70 mg/g FW). The maximum carotenoid content was likewise recorded in S_150_T_4_ (0.90 mg/g FW). Conversely, S_150_T_0_ yielded the lowest values across all photosynthetic pigments (chlorophyll a: 1.48, chlorophyll b: 0.44, total chlorophyll: 1.92, and carotenoid: 0.30 mg/g FW), with total carotenoid content being statistically similar to S_150_T_3_.

The chlorophyll a/b ratio exhibited a distinct trend compared to other pigment parameters. The highest chlorophyll a/b ratio was observed in S_0_T_3_ (3.19) under non-saline conditions and in S_150_T_3_ (3.34) under 150 mM NaCl. The lowest ratio was noted in S_0_T_4_ (2.75), which was numerically the lowest value but remained statistically similar to S_0_T_1_ and S_150_T_4_.

### 2.5. Correlation Analysis of Morphological, Physiological, and Biochemical Parameters

**Figure 4:**
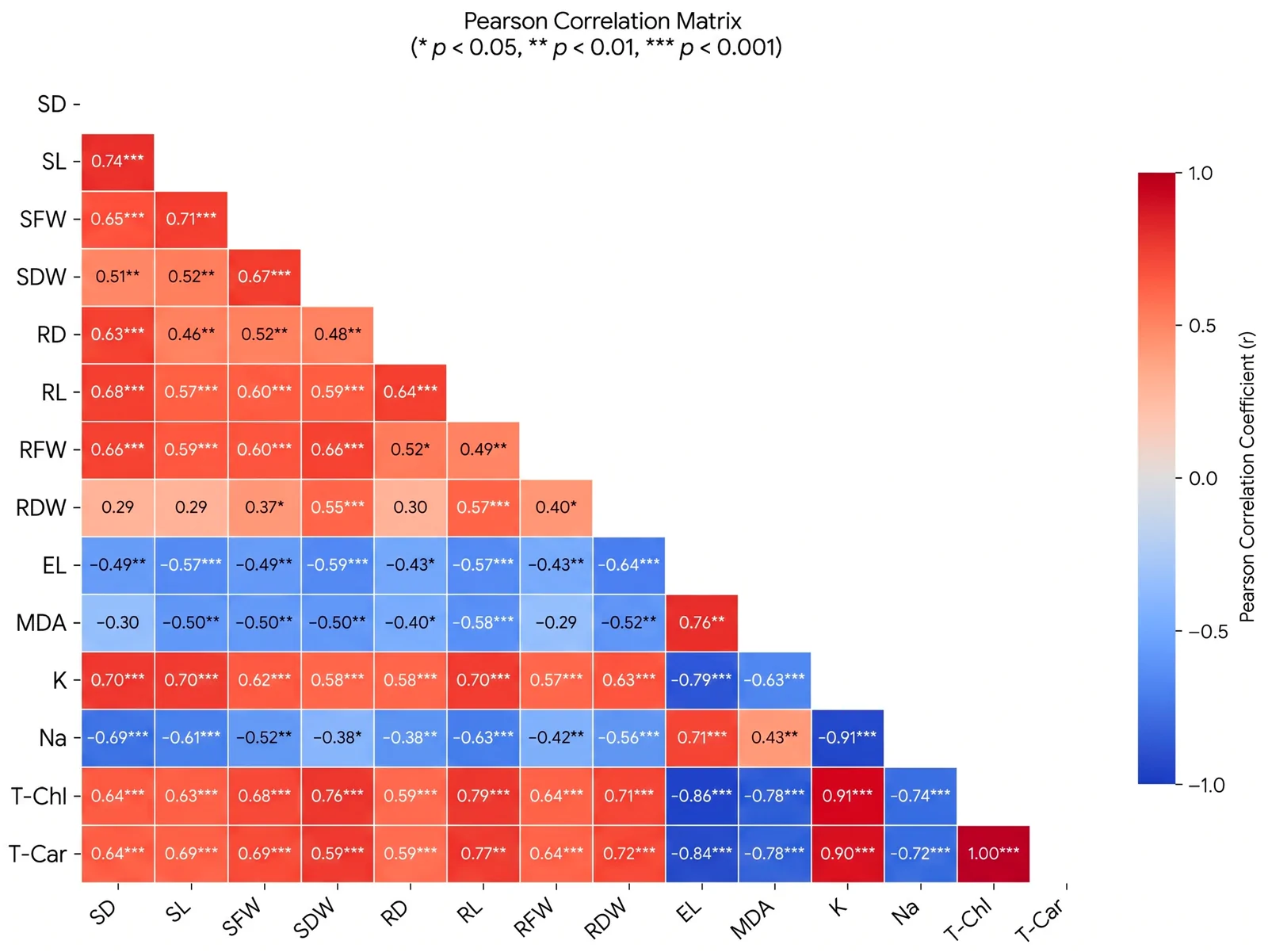
Heatmap of Pearson correlation analysis among morphological, physiological, and biochemical traits. Red indicates positive correlation, while blue indicates negative correlation. SD: stem diameter; SL: shoot length; SFW: shoot fresh weight; SDW: shoot dry weight; RD: root diameter; RL: root length; RFW: root fresh weight; RDW: root dry weight; EL: electrolyte leakage; MDA: malondialdehyde; K: potassium; Na: sodium; T-Chl: total chlorophyll; T-Car: total carotenoids.

Pearson correlation analysis revealed distinct relational patterns among growth parameters, oxidative stress markers, ion accumulation, and photosynthetic pigments (Figure 4). Growth and biomass traits including shoot length (SL), stem diameter (SD), root length (RL), root diameter (RD), and fresh/dry weights of shoots (SFW, SDW), and roots (RFW, RDW), demonstrated consistent positive correlations among themselves (r = 0.27 to 0.74, mostly *p* < 0.01). Similarly, photosynthetic pigments (total chlorophyll, T-Chl and total carotenoids, T-Car) and tissue potassium (K^+^) content exhibited strong positive relationships with vegetative growth attributes. In contrast, cellular damage indicators like EL and MDA along with Na^+^ accumulation, displayed marked negative correlations with growth traits and photosynthetic pigments. Specifically, EL and Na^+^ showed pronounced negative associations with T-Chl, T-Car, and K^+^ (r = −0.69 to −0.91). Furthermore, tissue Na^+^ content was positively associated with both EL and MDA (r = 0.43 to 0.71), whereas K^+^ was strongly and inversely correlated with Na^+^ (r = −0.91) and membrane damage markers (r = −0.63 to −0.79).

### 2.6. Principal Component Analysis (PCA)

**Figure 5:**
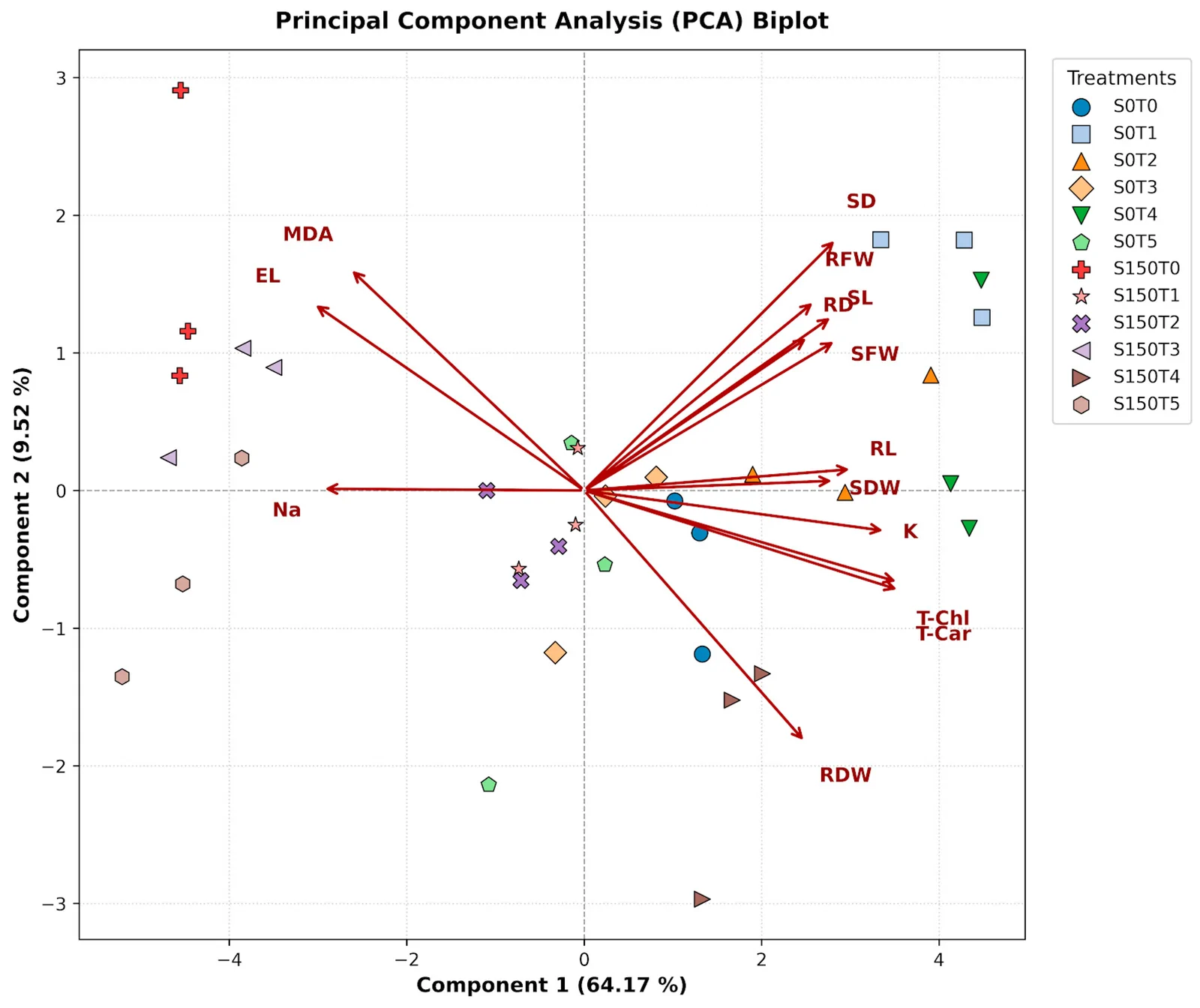
Principal component analysis (PCA) biplot displaying the multivariate relationships among morphological, physiological, and biochemical traits across different salinity levels (S_0_ and S_150_) and applied treatments (T_0_–T_5_). Vectors indicate evaluated parameters: shoot length (SL), root length (RL), stem diameter (SD), root diameter (RD), shoot fresh weight (SFW), shoot dry weight (SDW), root fresh weight (RFW), root dry weight (RDW), potassium content (K^+^), total chlorophyll (T-Chl), total carotenoids (T-Car), sodium content (Na^+^), electrolyte leakage (EL), and malondialdehyde (MDA).

Principal Component Analysis was performed to comprehensively evaluate the multivariate effects of different salinity levels (S_0_ and S_150_) and applied treatments (T_0_–T_5_) on the examined agronomic, physiological, and biochemical traits, as well as the relationships among these variables (Figure 5). The first two principal components accounted for 73.72% of the total variance, with Component 1 (PC_1_) and Component 2 (PC_2_) representing 64.2% and 9.52% of the variance, respectively. The PC_1_ axis clearly separated salt stress markers from growth and photosynthetic parameters. On the positive side of PC_1_, shoot length (SL), root length (RL), stem diameter (SD), root diameter (RD), shoot fresh weight (SFW), shoot dry weight (SDW), root fresh weight (RFW), root dry weight (RDW), potassium (K^+^), total chlorophyll (T-Chl), and total carotenoid (T-Car) concentrations clustered with strong positive correlations. Conversely, vectors for Na^+^ content, electrolyte leakage (EL), and malondialdehyde (MDA), which are indicators of salt toxicity and membrane damage, were located on the negative side of PC_1_. Regarding the distribution of treatment groups, control plants exposed to severe salt stress without protective treatment (S_150_T_0_) were situated in the upper-left quadrant (negative PC_1_, positive PC_2_), closely associated with high Na^+^, MDA, and EL levels. In contrast, non-salinized treatments generally grouped toward the central and positive PC_1_ region. Notably, certain protective treatments under salinity (particularly S_150_T_1_ and S_150_T_4_) displayed an evident shift away from the severe stress quadrant toward the origin and positive PC_1_/negative PC_2_ space, reflecting mitigated oxidative/membrane damage and partial recovery of growth and root-related traits.

## 3. Discussion

### 3.1. Effects of Salinity on Plant Growth and the Mitigating Role of Treatments

In the present study, exposure to 150 mM NaCl resulted in an overall suppression of shoot and root growth in faba bean plants. The significant decreases in SL, SFW, RL, and RDW observed in the S_150_T_0_ group relative to the non-saline control (S_0_T_0_) are consistent with the well-documented detrimental effects of salinity on vegetative growth [23]. Osmotic stress and disrupted ion homeostasis triggered under saline conditions severely restrict cell expansion and biomass accumulation across diverse plant taxa, particularly in glycophytic legumes [24,25,26]. Studies on faba bean further confirm that escalating NaCl levels depress shoot and root elongation, diminish fresh and dry biomass, and undermine photosynthetic performance [27,28,29].

Bacterial inoculation contributed substantially to mitigating salinity-induced growth inhibition. Shoot length reached 41.89 cm in the S_150_T_1_ treatment, compared with only 35.90 cm in the non-inoculated saline control (S_150_T_0_). These findings align with earlier reports showing that bacterial inoculation enhances root and shoot elongation alongside fresh and dry biomass production under salt stress [30,31,32]. Although endogenous ethylene levels were not directly quantified in the present study, the qualitative ACC deaminase capability previously identified in *Paenibacillus xylanilyticus* KF63C suggests that the mitigation of growth suppression may be associated with reduced stress-induced ethylene accumulation, facilitating more balanced vegetative development [30,33]. Furthermore, beneficial rhizobacteria have been shown to enhance salinity tolerance and restructure root–rhizosphere microbial dynamics in faba bean [11,13].

Nevertheless, solitary bacterial inoculation did not maximize performance across all growth parameters. Under non-saline conditions, while the S_0_T_1_ treatment promoted superior SD, SL, and SFW and RFW, the S_0_T_4_ treatment yielded higher values for RD, RL, and SDW. Interestingly, while S_0_T_4_ markedly stimulated root morphology, it exhibited a suppressive effect on shoot elongation. This dichotomy underscores that distinct biostimulant treatments can elicit differential, organ-specific morphological responses. The efficacy of PGPR and organic amendments is known to vary as a function of bacterial strain, host genotype, stress severity, and specific ambient regimes [11,13,31].

Notably, under 150 mM NaCl conditions, the S_150_T_4_ treatment achieved superior SDW (0.434 g) and RDW (0.348 g). Conversely, the decline of these parameters to 0.353 g and 0.193 g in the S_150_T_5_ treatment indicates that escalating the WV concentration does not necessarily yield proportional physiological benefits. This marked contrast delineates a critical concentration cut-off point between bio stimulation and phytotoxic inhibition. Pyroligneous acid represents a multi-component organic matrix whose bioactivity is strictly dose-dependent. While low concentrations (0.50% WV) serve as signaling triggers to stimulate metabolic acclimation and reinforce endogenous defenses, higher application rates (1.00% WV) introduce an excessive chemical burden onto the foliar surface. Specifically, the concentrated presence of low-molecular-weight organic acids (predominantly acetic acid, pH 5.49) lowers leaf boundary-layer pH, potentially eroding the epicuticular wax layer and compromising stomatal regulation [34]. The resulting stomatal closure limits internal carbon fixation and exacerbates non-stomatal limitations to photosynthetic efficiency. Concurrently, supra-optimal concentrations of exogenous volatile phenolics present in wood vinegar—particularly phenol, guaiacol, and syringol—can act as pro-oxidants rather than free radical scavengers. This phenolic overload triggers secondary oxidative perturbations, damages chloroplast envelope integrity, and impairs metabolic enzyme coordination in salt-stressed tissues. Consequently, exceeding the optimal biostimulatory window (0.50%) induces localized phytotoxicity that negates the therapeutic synergy otherwise achieved with rhizobacterial inoculation.

This outcome corroborates previous findings highlighting the critical role of concentration thresholds in WV applications, where lower concentrations effectively bolstered vigor in salt-stressed cucumber (*Cucumis sativus*) seedlings, whereas higher doses failed to confer comparable protection [34,35]. Nonetheless, this conclusion remains constrained by the two concentrations tested under the specific environmental conditions of the present experiment. Furthermore, it should be emphasized that the present investigation was conducted as a 30-day pot experiment under controlled growth-chamber conditions, focusing exclusively on early vegetative seedling growth. Consequently, these findings should be regarded as preliminary and cannot be directly interpreted as conclusive evidence of mature plant performance, pod formation, or field-level crop productivity. Comprehensive multi-year agronomic trials under diverse open-field and greenhouse conditions will be necessary to substantiate and validate these protective responses across full growing cycles.

These integrated morphological responses are strongly supported by both the Pearson correlation heatmap and the PCA biplot. According to the correlation heatmap (Figure 4), all vegetative growth and biomass traits demonstrated consistent and robust positive correlations among themselves (r = 0.55 to 0.95). In the multivariate space of the PCA biplot (Figure 5), these traits clustered tightly along the positive axis of Component 1 (PC1). Particularly, while the unamended salt stress control plants (S_150_T_0_) were projected into the upper-left quadrant associated with high damage indicators, the combined treatment under salinity (S_150_T_4_: PGPR + 0.50% WV) exhibited a distinct shift toward the positive PC1 coordinates and the growth vector cluster. This dual statistical validation confirms that the synchronized application of bacterial inoculation and low-dose WV effectively preserves macro-level seedling growth performance under 150 mM NaCl salinity.

### 3.2. Electrolyte Leakage and Malondialdehyde Content

Salinity-induced membrane deterioration and lipid peroxidation were clearly reflected in the EL and MDA dynamics. The marked elevation of EL to 57.24% and MDA content to 0.522 µg TEP equivalent/mL in the S150T0 treatment highlights the extent of oxidative membrane damage imposed by 150 mM NaCl. Salt stress regularly intensifies EL and lipid peroxidation across plant species [36,37,38]. In faba bean specifically, NaCl exposure triggers acute oxidative stress, membrane destabilization, and accelerated lipid peroxidation [27,39,40].

Both bacterial inoculation and WV co-application significantly modified these cellular damage markers. The S_150_T_4_ combination recorded the lowest membrane perturbation, with EL dropping to 19.26% and MDA content decreasing to 0.212 µg TEP equivalent/mL values remarkably lower than those of the unamended saline control (S_150_T_0_). These reductions correspond with reports demonstrating that PGPR inoculation limits EL and curtail MDA accumulation in salt-stressed tissues [30,41].

A comparable protective trend is documented for WV. Foliar application of WV attenuated oxidative injury, membrane permeability, and lipid peroxidation in salt-stressed rapeseed (*Brassica napus*) [42] and cucumber seedlings [34]. Moreover, fluctuations in tissue MDA and hydrogen peroxide (H_2_O_2_) levels are known to depend strictly on the applied pyroligneous extract concentration [43,44].

The marked decline in both EL and MDA levels in the S_150_T_4_ group suggests that this specific treatment effectively preserves membrane integrity and mitigates lipid peroxidation under severe salt stress. The coordinated attenuation of these two parameters implies that cellular membranes maintained structural stability, thereby minimizing secondary oxidative lesions induced by 150 mM NaCl. However, it should be emphasized that these measurements reflect physiological damage indicators rather than direct mechanistic proof of enzymatic antioxidant stimulation. Because the activities of specific antioxidant enzymes (e.g., superoxide dismutase, catalase, ascorbate peroxidase) and related gene expression levels were not directly assayed in this study, the observed reductions in EL and MDA are consistent with mitigated membrane perturbation, but do not independently establish the precise biochemical pathway responsible.

The direct physiological constraints of such membrane deterioration on plant development are prominently captured by both correlation and ordination analyses. The correlation heatmap (Figure 4) revealed that cellular damage indicators, namely EL and MDA, displayed highly pronounced negative associations (r = −0.60 to −0.85) with all shoot and root growth attributes. Mirroring these trends, the PCA biplot (Figure 5) positioned the EL and MDA vectors on the exact opposite (negative) side of the PC1 axis, completely separated from the vegetative biomass variables. The fact that the S_150_T_4_ group maintained the lowest coordinates along these damage vectors in the PCA matrix mathematically confirms that this specific formulation mitigates salinity-induced lipid peroxidation, thereby shielding the cellular membranes from oxidative degradation.

### 3.3. Potassium, Sodium Accumulation and K^+^/Na^+^ Ratio

Salinity stress substantially disrupted the elemental balance of faba bean tissues. In the S_150_T_0_ group, K^+^ content dropped to 1.21%, while Na^+^ content rose to 1.285%, depressing the K^+^/Na^+^ ratio to 0.94. Enhanced intracellular Na^+^ accumulation coupled with competitive inhibition of K^+^ uptake represents a classic hallmark of disrupted ion homeostasis under salt stress [24,45,46]. In faba bean, elevated cytosolic concentrations of Na^+^ and Cl^−^ severely hinder K^+^ and Ca^2+^ nutrition, leading to impaired metabolic activity, stunted growth, and reduced photosynthetic performance [28,33].

Among the salt-stressed groups, S_150_T_4_ exhibited the highest K^+^ accumulation (2.83%) and the lowest Na^+^ accumulation (0.646%), resulting in a superior K^+^/Na^+^ ratio of 4.40. In comparison, the solitary bacterial treatment (S_150_T_1_) established a K^+^/Na^+^ ratio of 2.53. These findings demonstrate that both PGPR alone and in combination with 0.50% WV foster a more favorable cellular ionic environment than the untreated saline control.

Bacterial inoculation has been extensively shown to facilitate K^+^ uptake, while restricting root-to-shoot Na^+^ translocation under salt stress [35,41,47]. In particular, PGPR applications in faba bean have been reported to restore K^+^/Na^+^ homeostasis, upregulate high-affinity potassium transporters, and support balanced mineral nutrition [34].

Concurrently, WV has been shown to restrict Na^+^ uptake, and assist in maintaining cellular ion equilibrium in salt-stressed rapeseed [42]. In saline-alkaline wheat fields, suitable pyroligneous acid amendments markedly enhanced K^+^ retention and depressed Na^+^ accumulation, thereby optimizing the cytosolic K^+^/Na^+^ balance [48]. Such physiological preservation is strongly supported by the beneficial plant–soil stress buffering capacity and ion-retention properties of organic matrices and bio-amendments, which regulate nutrient dynamics, decrease undesirable adsorption, and mitigate stress hazards in arid and calcareous media [49]. Because transcriptional profiles of ion transporter genes (such as SOS1 or HKT family transporters) and direct membrane transport assays were not evaluated in the present study, the enhanced K^+^/Na^+^ ratio cannot be unequivocally attributed to a specific molecular transport pathway. Rather, the empirical data demonstrate that the S_150_T_4_ treatment preserves tissue ion equilibrium at the macroscopic level, which is consistent with, but does not definitively prove, altered transport kinetics or exclusion mechanisms across root and shoot membranes.

This intracellular elemental antagonism is robustly illustrated through the combination of the correlation heatmap and the PCA model. The correlation heatmap (Figure 4) indicated a strong positive association of tissue Na^+^ with both EL and MDA (r = 0.50 to 0.75), whereas K^+^ was inversely correlated with Na^+^ (r = −0.75) and membrane damage markers. In the PCA biplot (Figure 5), the Na^+^ vector was oriented in a diametrically opposed direction relative to the growth attributes, reinforcing its toxic role. Conversely, the S_150_T_4_ application shifted dramatically away from the severe stress quadrant (S_150_T_0_) and clustered directly alongside the K^+^ content and K^+^/Na^+^ ratio vectors, empirically demonstrating that this combination effectively up hauls homeostatic ion regulation under severe salt stress.

### 3.4. Photosynthetic Pigments: Chlorophyll and Carotenoid Dynamics

Salinity stress provoked a substantial depletion of photosynthetic pigments. In S_150_T_0_ plants, chlorophyll a content declined to 1.48 mg/g FW, and chlorophyll b fell to 0.44 mg/g FW, compared with 3.18 mg/g FW and 1.08 mg/g FW in non-stressed control (S_0_T_0_), respectively. Total chlorophyll and total carotenoid pools dropped correspondingly to 1.92 mg/g FW and 0.30 mg/g FW in S_150_T_0_, contrasting with 4.26 mg/g FW and 0.80 mg/g FW in S_0_T_0_. Salinity-induced degradation of photosynthetic pigments and suppression of their de novo biosynthesis are widely documented in faba bean and related legumes [27,28,48].

Among all treatments evaluated, the S_150_T_4_ combination stood out prominently. In this group, chlorophyll a content reached 3.48 mg/g FW, surpassing all other saline treatments and exceeding the level recorded in the non-stressed control (S_0_T_0_). Chlorophyll b, total chlorophyll, and total carotenoids reached 1.22 mg/g FW, 4.70 mg/g FW, and 0.90 mg/g FW, respectively, representing the highest absolute pigment con-tents recorded under salinity. Furthermore, the chlorophyll a/b ratio, which rose to 3.35 under untreated salt stress (S_150_T_0_), was restored to near-basal levels (2.86) in the S_150_T_4_ group, indicating enhanced structural stabilization of the light-harvesting pigment–protein complexes against salt-induced photo-oxidative degradation. While higher total chlorophyll and carotenoid contents in S_150_T_4_ indicate better pigment retention un-der salinity, these results represent gross tissue pigment pools. Ultrastructural preservation of thylakoid membranes or direct protection of chloroplast protein complexes was not microscopically or molecularly verified; thus, the elevated pigment levels should be interpreted as physiological markers of reduced photo-oxidative breakdown rather than direct evidence of molecular complex stabilization.

These results corroborate earlier evidence that optimal WV supplementation protects the photosynthetic apparatus under saline conditions. In salt-stressed cucumber seedlings, WV promoted chlorophyll a and b synthesis, and maintained elevated carotenoid pools [14]. Similar preservation of photosystem II efficiency and photosynthetic pigment complexes via pyroligneous acid has been documented in rapeseed [42].

However, escalating the WV concentration did not confer proportional photoprotection. In the S_150_T_5_ group (1.0% WV), chlorophyll a and b contents remained at 1.86 mg/g FW and 0.61 mg/g FW, while total chlorophyll and carotenoids dropped to 2.47 mg/g FW and 0.41 mg/g FW, respectively, substantially lower than the levels maintained by S_150_T_4_. This pattern confirms that excessive doses of pyroligneous acid do not enhance pigment retention, and may exert mild phytotoxic constraints, consistent with previous dose-dependency studies in cucumber and wheat [34,48]. This dose-dependent decline in pigment retention under the 1.00% WV treatment (S_150_T_5_) can be mechanistically linked to the chemical profile of the extract. The excessive accumulation of exogenous phenolic compounds (such as phenol, cresol, and pyrocatechol) on the foliar surface at higher doses can disrupt the cellular redox balance and trigger localized osmotic or chemical stress. This structural overloading likely compromises the integrity of the chloroplast envelopes, and accelerates the degradation of light-harvesting pigment–protein complexes, thereby diminishing the photoprotective capacity of the biostimulant.

Concurrently, the holistic integration of the photosynthetic apparatus with the overall physiological state is neatly summarized by the correlation heatmap and PCA matrix. The correlation heatmap (Figure 4) unveiled a highly synchronized positive relationship between T-Chl, (T-Car, and tissue K^+^ content, confirming that ion balance is a critical prerequisite for safeguarding chloroplast integrity against salt damage. In the PCA biplot (Figure 5), the pigment vectors clustered closely on the positive side of PC_1_, where the S_150_T_4_ group outperformed all other saline treatments by aligning closest to these vectors. This dual evidence substantiates that a 0.50% WV co-application operates within an optimal biostimulatory window to protect photosystem complexes, whereas exceeding this threshold (S_150_T_5_: 1.0% WV) triggers minor phytotoxic constraints that are explicitly captured as a backward shift on the PC_1_ axis.

## 4. Materials and Methods

### 4.1. Plant Material and Experimental Setup

Certified broad bean seeds registered by the Aegean Agricultural Research Institute (AARI), İzmir, Türkiye, were utilized in this study. The experiment was conducted as a pot trial for 30 days in a controlled walk-in growth room located at the Department of Field Crops, Faculty of Agriculture, Siirt University, Türkiye. Walk-in growth room conditions were adjusted to a constant temperature of 25 ± 3 °C, a relative humidity of 45–55%, and a 16 h light/8 h dark photoperiod. Prior to sowing, uniform healthy seeds were surface-sterilized by immersion in 70% (*v*/*v*) ethanol for 1 min, followed by 2% (*w*/*v*) sodium hypochlorite (NaOCl) for 5 min, and thoroughly rinsed five times with sterile distilled water. Plastic pots with a capacity of 3 L (15 × 15 × 20 cm) featuring perforated bases and equipped with individual saucers were filled with a sterile substrate mixture of commercial white *Sphagnum* peat moss and agricultural perlite (2:1, *v*/*v*). The growing medium was characterized by an adjusted slightly acidic to neutral pH of 6.0, a low baseline electrical conductivity (EC) of 0.35 dS m^−1^ an organic matter content > 80%, and a dry bulk density of approximately 0.15 g cm^−3^. Ten seeds were sown per pot, and following emergence, seedlings were thinned to seven uniform, healthy plants per pot. Substrate field capacity was determined gravimetrically prior to sowing. To regulate the leaching fraction and prevent uncontrolled salt accumulation or drainage loss in the rhizosphere, moisture was maintained at 80% of field capacity by gravimetrically weighing the pots every two days throughout the 30-day experimental trial. Any minimal drainage collected in individual saucers was retained and reabsorbed via capillary action, preventing nutrient loss and cross-contamination among treatments. Substrate EC was periodically verified to monitor the target salinity regime without erratic salt build-up.

### 4.2. Production and Chemical Characterization of Pistachio Hull Wood Vinegar

The WV used in this study was produced by the controlled slow pyrolysis of fresh fruit hulls of Siirt pistachio (*Pistacia vera* L. cv. Siirt) in a custom-built, oxygen-limited reactor at 350 °C with a heating rate of 5–10 °C min^−1^ and a 2 h residence time. The fundamental physicochemical properties (pH, electrical conductivity) and available mineral nutrient profile of the WV were determined at the accredited laboratories of the Environmental Protection Department of Istanbul Metropolitan Municipality (IMM), Türkiye. The volatile organic chemical composition was analyzed using a gas chromatography–mass spectrometry system (GC-MS, ISQ Series, ISQ LT, Thermo Fisher Scientific, Waltham, MA, USA) at the Siirt University Science and Technology Application and Research Center, and peak identities were confirmed using NIST and Wiley (Wiley9, WILEY7, mainlib) reference spectral libraries. The physicochemical attributes and predominant bioactive compounds identified in the WV are summarized in Table 2.

### 4.3. Bacterial Strain, Inoculum Preparation, and Seed Inoculation

In this study, *Paenibacillus xylanilyticus* strain KF63C, obtained as a pre-identified gift culture from the Department of Field Crops, Faculty of Agriculture, Siirt University, Türkiye, was utilized as an asymbiotic PGPR. This specific isolate was selected for the present study based on its pre-determined, multifaceted plant growth-promoting (PGP) traits, where its taxonomic status had been previously established via morphological, biochemical, and molecular identification procedures by the provider institution. The in vitro PGP attributes of strain KF63C including nitrogen fixation capacity, phosphate solubilization, ACC deaminase activity, and siderophore production had been characterized via standard qualitative screening assays by the providing institution, as summarized in Table 3. The strain exhibited multiple concurrent PGP mechanisms, showing a positive reaction for nitrogen fixation and siderophore production (+), and moderate activity (++) for both phosphate solubilization and ACC deaminase activity. Because the primary focus of the current research was to evaluate the in vivo agronomic impacts of the bacterium rather than re-characterizing its in vitro biochemical pathways, these pre-determined qualitative traits were adopted as baseline screening data. For the experiments, the bacterial stock culture was initially inoculated onto a solid culture medium containing 20 g L^−1^ Nutrient Agar and incubated at 30 °C for 24 h to obtain single colonies. Subsequently, well-isolated single colonies were transferred into 8 g L^−1^ Nutrient Broth liquid medium. The liquid culture was incubated in an orbital shaker at 28 °C and 150 rpm for 48 h until the bacterial population reached its optimal logarithmic growth phase. The cell density of the suspension was adjusted spectrophotometrically to an optical density of OD_600_ = 0.8, yielding a standard bacterial suspension of approximately 1.0 × 10^8^ CFU mL^−1^ [51]. Broad bean seeds, which had been surface-sterilized, were completely immersed in the prepared bacterial suspension to ensure a homogenous coating of the seed coat with the PGPR inoculum. For the non-inoculated control treatment, seeds were immersed in sterile Nutrient Broth for the exact same duration to eliminate any bias arising solely from the nutrient medium. Following the immersion process, all treated seeds were air-dried in the shade within a sterile laminar flow hood to prevent desiccation and sown within 1 h of application, while being strictly protected from direct sunlight to preserve bacterial viability against ultraviolet radiation. To confirm that the inoculated bacterial strain successfully persisted in the root zone under severe salinity throughout the 30-day experimental period, rhizospheric substrate and root-surface washings were qualitatively evaluated at harvest. Aliquots were plated onto Nutrient Agar, and persistent bacterial viability and characteristic colony recovery corresponding to the inoculated *Paenibacillus* strain were verified in inoculated treatments, while remaining absent in non-inoculated controls.

### 4.4. Experimental Setup, Treatments, and Salinity Stress Induction

The experiment was conducted using a factorial design based on a completely randomized design (CRD) with two salinity levels and six protective treatments, each replicated three times, in 3-L pots. The experimental factors comprised two salinity levels (S_0_: 0 mM NaCl and S_150_: 150 mM NaCl) and six protective treatments (T_0_: Control; T_1_: PGPR; T_2_: 0.50% WV; T_3_: 1.00% WV; T_4_: PGPR + 0.50% WV; T_5_: PGPR + 1.00% WV). Salinity stress was imposed gradually with the irrigation water until the 150 mM NaCl concentration was achieved. The first foliar application of WV solutions was performed when the broad bean seedlings reached the 3–4 true-leaf stage. Subsequent foliar applications were systematically repeated twice a week throughout the remainder of the 30-day experimental period. WV working solutions (0.50% and 1.00%, *v*/*v*) were prepared by diluting with distilled water and sprayed onto foliar surfaces using a hand-held pressure sprayer until run-off, while non-WV-treated control plants were sprayed with an equal volume of distilled water at the same intervals.

### 4.5. Determination of Morphological Parameters

On day 30 of the experiment, five randomly selected, homogeneously developed plants per pot were harvested for morphological analyses to evaluate shoot and root growth. To assess plant development, shoot length, stem diameter, shoot fresh weight, shoot dry weight, root length, root diameter, root fresh weight, and root dry weight were determined. For all measured parameters, the mean value of these five plants was recorded as a single replicate value.

Shoot length was determined by measuring the distance between the soil surface and the uppermost growing point of the shoot using a precision ruler (±0.1 cm). Stem diameter was measured at a predetermined point 2 cm above the soil surface using a digital caliper (±0.01 mm; Model 500-182-30, Mitutoyo, Kawasaki, Japan). Because the stem possessed a non-circular geometry, two mutually perpendicular measurements were taken at the same point, and their arithmetic mean was recorded.

During harvest, plants were carefully removed from the pots to prevent damage to the root system. The roots were gently washed with tap water until all adhering soil particles were completely removed. The cleaned roots were placed on a flat surface, and the distance from the root-shoot junction to the tip of the primary root was measured using a precision ruler (±0.1 cm). Following root cleaning, root diameter was measured at a predetermined region of the primary root adjacent to the root-shoot junction using the same digital caliper, taking care not to compress the root tissue; values were recorded in millimeters.

Harvested plants were then separated into shoot and root fractions. Surface water on the shoot and root tissues was gently blotted dry using paper towels without damaging the tissue integrity. Subsequently, shoot and root samples were weighed using an analytical balance (±0.0001 g)(AS 220.R2, RADWAG, Radom, Poland), and the obtained values were recorded as shoot fresh weight and root fresh weight, respectively. Results were expressed as g plant^−1^. Plant tissues were then dried in a forced-air oven (UN160, Memmert GmbH + Co. KG, Schwabach, Germany) at 65 °C for 72 h until reaching a constant weight. After drying, samples were allowed to cool to room temperature and reweighed using the same analytical balance to determine shoot dry weight and root dry weight.

### 4.6. Physiological and Biochemical Assays

#### 4.6.1. Electrolyte Leakage

Membrane permeability was determined using the electrolyte leakage (EL) method. Fully expanded leaf discs (10 uniform discs of 1.0 cm diameter) were thoroughly rinsed with deionized water to remove surface-adhering electrolytes from cut cell walls and gently blotted dry with filter paper. The discs were then placed in test tubes containing 10 mL of deionized water and incubated (UN160, Memmert GmbH + Co. KG, Schwabach, Germany) at 25 °C for 24 h to record the initial electrical conductivity (EC_1_) using an EC meter (Orion Star A212, Thermo Fisher Scientific Inc., Waltham, MA, USA). Subsequently, the tubes were autoclaved (VAPOUR-Line, VWR International, Radnor, PA, USA) at 121 °C for 20 min to induce complete tissue rupture, cooled to 25 °C, and the final conductivity (EC_2_) was recorded. Membrane injury was calculated using the following equation:

EL (%) = (EC_1_/EC_2_) × 100.

#### 4.6.2. Malondialdehyde (MDA) Content

Lipid peroxidation was determined using the thiobarbituric acid (TBA) assay [52,53]. Frozen leaf tissues stored at −80 °C were weighed and homogenized in an ice-cold mortar with 0.1% (*w*/*v*) trichloroacetic acid (TCA) at a 1:10 (*w*/*v*) fresh tissue-to-buffer ratio and centrifuged at 10,000 rpm (4 °C) for 20 min (Megafuge 16R, Thermo Scientific, Waltham, MA, USA). An aliquot of 1 mL supernatant was mixed with 2 mL of 0.5% (*w*/*v*) TBA prepared in 20% (*w*/*v*) TCA. The mixture was incubated at 95 °C for 30 min in a water bath, immediately cooled in an ice bath to terminate the reaction, and centrifuged at 5000 rpm for 10 min. Absorbance values were measured at 532 and 600 nm on a microplate spectrophotometer (Multiskan GO, Thermo Fisher Scientific, Vantaa, Finland). Nonspecific turbidity (A_532_ − A_600_) was subtracted, and MDA concentrations were calculated using a 1,1,3,3-tetraethoxypropane (TEP) standard curve (y = 0.214x + 0.018, R^2^ = 0.9995). The obtained values were normalized considering the 1:10 (*w*/*v*) extraction ratio and expressed on a fresh weight (FW) basis as µg TEP equiv. mL^−1^ extract.

#### 4.6.3. Chlorophyll and Carotenoid Analysis

Photosynthetic pigments were extracted and quantified spectrophotometrically according to the method of Arnon [54] with minor modifications. Briefly, 0.1 g of fully expanded fresh leaf tissue was homogenized in 10 mL of 80% (*v*/*v*) ice-cold aqueous acetone in the dark. The homogenate was centrifuged at 4000 rpm for 10 min at 4 °C. The absorbance of the clear supernatant was determined at 663 nm (A_663_), 645 nm (A_645_), and 470 nm (A_470_) against an 80% acetone blank using a UV-Vis spectrophotometer (Evolution 201, Thermo Scientific, Waltham, MA, USA). Concentrations of chlorophyll a (Chl a), chlorophyll b (Chl b), total chlorophyll (Total Chl), and total carotenoids (Total Car) were calculated using the following equations and expressed as mg g^−1^ fresh weight (FW):

Chlorophyll a (mg g^−1^ FW) = [(12.7 × A_663_) − (2.69 × A_645_)] × [V/(1000 × W)]

Chlorophyll b (mg g^−1^ FW) = [(22.9 × A_645_) − (4.68 × A_663_)] × [V/(1000 × W)]

Total Chlorophyll (mg g^−1^ FW) = [(20.2 × A_645_) + (8.02 × A_663_)] × [V/(1000 × W)]

Total Carotenoids (mg g^−1^ FW) = [(1000 × A_470_ − 1.82 × Chl a − 85.02 × Chl b)/198] × [V/(1000 × W)]

#### 4.6.4. Mineral Analysis

Dried and ground leaf samples (0.5 g) were transferred to Teflon vessels with 10 mL of 65% HNO_3_ and 2 mL of 30% H_2_O_2_. Samples were digested in a microwave digestion system (Speedwave MWS-2, Berghof Products + Instruments GmbH, Eningen, Germany) using a three-stage program (145 °C for 10 min, 160 °C for 10 min, and 190 °C for 20 min at 80% power) [55]. Cooled digests were diluted to 50 mL with deionized water. Na^+^ and K^+^ concentrations were measured on a flame photometer (PFP7, Jenway, Cole-Parmer, Stone, UK) calibrated with standard solutions. Element contents were calculated on a dry weight percentage (%) basis, and the K^+^/Na^+^ ratio was computed.

### 4.7. Statistical Analysis

Data were subjected to a two-way analysis of variance (ANOVA) following a factorial completely randomized design (two salinity levels × six protective treatments) using JMP Student Edition (Version 19, JMP Statistical Discovery LLC, Cary, NC, USA). The main effects of salinity and protective treatments, as well as their two-way interaction (Salinity × Treatment), were evaluated. Significant differences among treatment means were determined using Tukey’s Honestly Significant Difference (HSD) post-hoc test, with statistical significance declared at *p* < 0.05 and exact *p*-values reported where applicable [56].

## 5. Conclusions

The collective findings demonstrate that 150 mM NaCl stress significantly retards vegetative growth, exacerbates membrane permeability and lipid peroxidation, disrupts cellular K^+^/Na^+^ homeostasis, and degrades photosynthetic pigments in faba bean (*Vicia faba* L.). Bacterial inoculation alone partially mitigated these inhibitory effects, notably enhancing shoot elongation. However, bacterial inoculation alone did not uniformly maximize all evaluated physiological indices. Under non-saline conditions, S_0_T_1_ promoted superior shoot elongation and fresh biomass, whereas S_0_T_4_ favored root morphological development and pigment pools while moderately restricting shoot elongation.

The most pronounced mitigation under salinity stress was achieved with the combined application of PGPR (*Paenibacillus xylanilyticus* KF63C) and 0.50% WV (S_150_T_4_). This co-treatment distinctly minimized cellular injury (yielding the lowest EL and MDA levels), maintained tissue ionic equilibrium (highest K^+^ accumulation and K^+^/Na^+^ ratio), and preserved the photosynthetic machinery (highest chlorophyll and carotenoid contents alongside a normalized chlorophyll a/b ratio). Furthermore, the marked contrast between S_150_T_4_ and S_150_T_5_ confirms that pyroligneous acid exhibits a concentration-dependent threshold, wherein the 0.50% dose represents an optimal biostimulatory window, while the 1.00% dose exerts minor phytotoxic constraints.

Nevertheless, because these findings were obtained from a 30-day pot trial conducted under controlled growth-chamber conditions, they should be considered preliminary and do not establish proven field-level effectiveness, mature-plant reproductive performance, or final seed yield. Further multi-season greenhouse and open-field trials are warranted to validate the agronomic sustainability, underlying molecular pathways, and practical viability of this combined biological approach under variable agro-ecological environments.

## Figures and Tables

**Table 1:** Two-way analysis of variance (ANOVA) of salinity (S), treatments (T), and their interaction (S × T) on growth, physiological, and biochemical parameters of broad bean (*Vicia faba* L.).

	*F*			*p*-Value				
Parameters	Salinity (S) (df = 1)	Treatment (T) (df = 5)	S × T Interaction (df = 5)	Salinity (S) (df = 1)	Treatment (T) (df = 5)	S × T Interaction (df = 5)	η_p_^2^ (S × T)	Error MS (df = 24)
Shoot Length (cm)	34.11	4.72	5.50	<0.0001	0.0038	0.0002	0.534	7.589
Stem Diameter (mm)	37.05	5.06	6.16	<0.0001	0.0026	<0.0001	0.562	0.061
Shoot Fresh Weight (g)	14.93	5.60	4.47	0.0007	0.0015	0.0011	0.482	0.357
Shoot Dry Weight (g)	7.55	13.67	8.63	0.0112	<0.0001	<0.0001	0.643	0.001
Root Length (cm)	40.21	19.18	13.52	<0.0001	<0.0001	<0.0001	0.738	1.582
Root Diameter (mm)	20.11	5.13	5.12	0.0002	0.0024	0.0004	0.516	0.055
Root Fresh Weight (g)	19.97	12.58	9.27	0.0002	<0.0001	<0.0001	0.659	0.215
Root Dry Weight (g)	8.33	4.21	3.54	0.0081	0.0069	0.0046	0.424	0.002
Electrolyte Leakage (%)	217.18	42.20	62.28	<0.0001	<0.0001	<0.0001	0.928	7.647
Malondialdehyde (µg TEP equiv. mL^−1^)	145.47	133.11	90.06	<0.0001	<0.0001	<0.0001	0.949	0.0003
Leaf K^+^ (% DW)	6632.27	324.46	780.94	<0.0001	<0.0001	<0.0001	0.994	0.004
Leaf Na^+^ (% DW)	6631.26	220.24	820.73	<0.0001	<0.0001	<0.0001	0.994	0.002
Leaf K^+^/Na^+^ Ratio	7464.48	85.59	761.29	<0.0001	<0.0001	<0.0001	0.994	0.383
Chlorophyll a (mg g^−1^ FW)	2391.70	574.35	525.03	<0.0001	<0.0001	<0.0001	0.991	0.003
Chlorophyll b (mg g^−1^ FW)	1193.65	382.72	305.97	<0.0001	<0.0001	<0.0001	0.985	0.001
Chlorophyll a/b Ratio	52.56	65.32	42.36	<0.0001	<0.0001	<0.0001	0.898	0.003
Total Chlorophyll (mg g^−1^ FW)	1951.31	508.06	445.90	<0.0001	<0.0001	<0.0001	0.989	0.008
Total Carotenoids (mg g^−1^ FW)	1101.38	320.31	266.65	<0.0001	<0.0001	<0.0001	0.982	0.001

Data represent the results of two-way ANOVA with three replicates (*n* = 3). df, degrees of freedom; MS, mean square; F, F-ratio; *p*, probability value; η_p_^2^, partial eta-squared (effect size for S × T interaction); S, salinity; T, treatment; DW, dry weight; FW, fresh weight; TEP, 1,1,3,3-tetraethoxypropane; K^+^, potassium; Na^+^, sodium.

**Table 2:** Physicochemical properties and major volatile organic compounds of pistachio fruit hull wood vinegar.

Parameter/Major Compound	Value/Area (%)	Classification/Method
pH (25 °C)	5.49	1:10 Potentiometric
Electrical Conductivity (EC)	3770 µS cm^−1^	1:10 Aqueous solution
Total Nitrogen (N)	0.84%	Kjeldahl method (ISO 11261) [50]
Available Ca/Na/K	25.35/5.32/3.39 mg kg^−1^	ICP-OES/Flame photometry
Available Mg/P/Fe	1.87/1.03/0.42 mg kg^−1^	Spectrophotometric & ICP-OES
Phenol	10.24%	Phenolics (RT: 5.52 min)
2-Methoxyphenol (Guaiacol)	7.43%	Methoxyphenols (RT: 8.85 min)
2,6-Dimethoxyphenol (Syringol)	5.97%	Methoxyphenols (RT: 19.27 min)
2-Cyclopenten-1-one	4.45%	Cyclopentenones (RT: 2.60 min)
2-Furanmethanol (Furfuryl alcohol)	4.35%	Furans (RT: 2.89 min)
3-Methyl-2-cyclopenten-1-one	4.27%	Cyclopentenones (RT: 5.00 min)
2,3-Dimethyl-2-cyclopenten-1-one	2.89%	Cyclopentenones (RT: 7.11 min)
2-Methyl-2-cyclopenten-1-one	2.49%	Cyclopentenones (RT: 3.75 min)
3-/4-Methylphenol (*m*/*p*-Cresol)	2.13%	Phenolics (RT: 8.46 min)
1,2-Benzenediol (Pyrocatechol)	1.69%	Diphenols (RT: 13.29 min)

Abbreviations: Ca: Calcium; Na: Sodium; K: Potassium; Mg: Magnesium; P: Phosphorus; Fe: Iron; RT: Retention Time; ICP-OES: Inductively Coupled Plasma Optical Emission Spectrometry; ISO 11261: International Organization for Standardization method for total nitrogen determination [50]. Area (%) represents the relative percentage of the compound peak area in the chromatographic profile.

**Table 3:** Qualitative plant growth-promoting (PGP) traits of *Paenibacillus xylanilyticus* strain KF63C.

Bacterial Code	Species	Nitrogen Fixation Capacity	Phosphate Solubilization Capacity	ACC Deaminase Activity	Siderophore Production
KF63C	*Paenibacillus xylanilyticus*	+	++	++	+

+: Positive reaction; ++: Moderate activity.

## Data Availability

The data are contained within the article.

## Supplementary Materials

The following supporting information can be downloaded at: https://www.mdpi.com/article/10.3390/plants15213360/s1, Table S1: Effects of seed-inoculated PGPR and foliar-applied WV on shoot growth parameters of broad bean (*Vicia faba* L.) under non-saline (0 mM NaCl) and saline (150 mM NaCl) conditions. Table S2: Effects of seed-inoculated PGPR and foliar-applied WV on root growth parameters of broad bean (*Vicia faba* L.) under non-saline (0 mM NaCl) and saline (150 mM NaCl) conditions. Table S3: Effects of seed-inoculated PGPR and foliar-applied WV on membrane permeability (EL), lipid peroxidation (MDA), and ion accumulation (K+, Na+, K^+^/Na^+^) of broad bean (*Vicia faba* L.) under non-saline (0 mM NaCl) and saline (150 mM NaCl) conditions. Table S4: Effects of seed-inoculated PGPR and foliar-applied WV on photosynthetic pigment contents (Chlorophyll a, b, total chlorophyll, total carotenoids) and chlorophyll a/b ratio of broad bean (*Vicia faba* L.) under non-saline (0 mM NaCl) and saline (150 mM NaCl) conditions.
