# Supplementary material for: Combined Effects of Seed-Inoculated PGPR and Foliar-Applied Pistachio Hull Wood Vinegar on Salinity Stress Alleviation in Broad Bean (Vicia faba L.)
Source: Plants (Basel). 2026 Oct 29;15(21):3360. doi: 10.3390/plants15213360 (PMC13765701; doi:10.3390/plants15213360)
Supplement: Supplementary file 1 [file plants-15-03360-s001.zip › plants-4644817-supplementary.pdf]

Supplemantary Tables

Table S1. Effects of seed-inoculated PGPR and foliar-applied WV on shoot growth parameters of broad bean (*Vicia faba* L.) under non-saline (0 mM NaCl) and saline (150 mM NaCl) conditions.

**Treatments**	**Shoot Diameter (mm)**	**Shoot Length (cm)**	**Shoot Fresh Weight (g)**	**Shoot Dry Weight (g)**
S_0T_0	3.45±0.10 a-d	41.78±0.69 a-c	5.529±0.904 bc	0.432±0.023 ab
S_0T_1	3.92±0.14 a	48.78±1.39 a	7.340±0.072 a	0.443±0.024 a
S_0T_2	3.74±0.07 a-c	46.11±0.84 ab	6.538±0.793 a-c	0.396±0.064 a-c
S_0T_3	3.25±0.02 a-d	42.89±4.03 a-c	6.049±0.793 a-c	0.358±0.044 bc
S_0T_4	3.78±0.22 ab	43.74±0.78 a-c	6.800±0.013 ab	0.474±0.012 a
S_0T_5	3.27±0.29 a-d	40.33±6.01 bc	5.366±0.982 bc	0.334±0.018 c
$S_{150}T_0$	3.16±0.26 b-d	35.90±0.67 c	5.070±0.490 bc	0.331±0.010 c
$S_{150}T_1$	3.21±0.42 a-d	41.89±0.49 a-c	5.502±0.274 bc	0.405±0.006 a-c
$S_{150}T_2$	3.13±0.09 b-d	39.11±0.38 bc	5.706±0.283 a-c	0.411±0.014 a-c
$S_{150}T_3$	3.01±0.01 cd	37.67±1.73 c	5.495±0.544 bc	0.349±0.020 c
$S_{150}T_4$	3.16±0.40 b-d	39.00±4.85 bc	6.287±0.290 a-c	0.434±0.005 ab
$S_{150}T_5$	2.67±0.38 d	37.89±2.81 c	4.944±0.695 c	0.353±0.032 bc
Tukey value/Significance	0.73**	8.11**	1.760**	0.083**

Values are means ± standard error (n = 3). In each column, means followed by the same lowercase letter are not significantly different according to Tukey's honestly significant difference (HSD) test. Tukey value indicates the HSD value at the 0.01 level (**, $p < 0.01$). S_0: 0 mM NaCl, S_{150}: 150 mM NaCl. T_0: Control, T_1: PGPR, T_2: 0.50% WV, T_3: 1.00% WV, T_4: PGPR + 0.50% WV, T_5: PGPR + 1.00% WV (PGPR: Plant growth-promoting rhizobacteria, *Paenibacillus xylanilyticus* strain KF63C; WV: Wood vinegar, also known as pyroligneous acid).

Table S2. Effects of seed-inoculated PGPR and foliar-applied WV on root growth parameters of broad bean (*Vicia faba* L.) under non-saline (0 mM NaCl) and saline (150 mM NaCl) conditions.

**Treatments**	**Root Diameter (mm)**	**Root Length (cm)**	**Root Fresh Weight (g)**	**Root Dry Weight (g)**
S_0T_0	2.51±0.24 bc	23.06±1.13 bc	4.108±0.622 bc	0.308±0.061 ab
S_0T_1	2.84±0.21 ab	24.71±1.76 ab	5.904±0.147 a	0.286±0.029 ab
S_0T_2	2.91±0.33 ab	27.86±1.27 a	4.165±0.383 bc	0.299±0.041 ab
S_0T_3	2.68±0.26 a-c	22.72±0.58 bc	3.210±0.273 c	0.240±0.033 ab
S_0T_4	3.26±0.49 a	28.01±0.46 a	4.622±0.215 ab	0.303±0.060 ab
S_0T_5	2.57±0.03 a-c	22.19±2.16 bc	3.333±0.327 bc	0.262±0.060 ab
$S_{150}T_0$	2.49±0.09 bc	21.38±0.42 bc	3.634±0.536 bc	0.197±0.084 b
$S_{150}T_1$	2.67±0.07 a-c	24.35±0.34 ab	3.768±0.513 bc	0.269±0.039 ab
$S_{150}T_2$	2.65±0.24 a-c	22.50±0.08 bc	4.020±0.447 bc	0.230±0.044 ab
$S_{150}T_3$	2.37±0.17 bc	20.25±0.24 c	2.878±0.678 c	0.183±0.008 b
$S_{150}T_4$	2.47±0.20 bc	24.76±1.75 ab	3.844±0.222 bc	0.348±0.044 a
$S_{150}T_5$	2.01±0.07 c	19.37±2.09 c	3.056±0.739 c	0.193±0.024 b
Tukey value/Significance	0.69**	3.70**	1.363**	0.141**

Values are means ± standard error (n = 3). In each column, means followed by the same lowercase letter are not significantly different according to Tukey's honestly significant difference (HSD) test. Tukey value indicates the HSD value at the 0.01 level (**, $p < 0.01$). S_0: 0 mM NaCl, S_{150}: 150 mM NaCl. T_0: Control, T_1: PGPR, T_2: 0.50% WV, T_3: 1.00% WV, T_4: PGPR + 0.50% WV, T_5: PGPR + 1.00% WV (PGPR: Plant growth-promoting rhizobacteria, *Paenibacillus xylanilyticus* strain KF63C; WV: Wood vinegar, also known as pyroligneous acid).

Table S3. Effects of seed-inoculated PGPR and foliar-applied WV on membrane permeability (EL), lipid peroxidation (MDA), and ion accumulation (K^+, Na^+, K^+/Na^+) of broad bean (*Vicia faba* L.) under non-saline (0 mM NaCl) and saline (150 mM NaCl) conditions.

**Treatments**	**Electrolyte Leakage (%)**	**MDA (µg TEP equiv./mL)**	**K^+ (% DW)**	**Na^+ (% DW)**	**K^+/Na^+ Ratio**
S_0T_0	19.19±0.60 c	0.388±0.009 c	3.48±0.07 b	0.130±0.005 f	26.85±0.96 a
S_0T_1	21.61±6.26 c	0.295±0.009 de	3.73±0.08 a	0.220±0.009 f	16.98±0.89 c
S_0T_2	21.22±1.82 c	0.242±0.013 fg	3.39±0.04 b	0.190±0.008 f	17.81±0.65 c
S_0T_3	26.31±0.94 c	0.254±0.009 e-g	3.09±0.04 c	0.228±0.009 f	13.59±0.61 d
S_0T_4	25.30±3.79 c	0.230±0.009 g	3.85±0.07 a	0.189±0.008 f	20.36±1.07 b
S_0T_5	22.71±3.16 c	0.308±0.043 d	3.07±0.05 c	0.139±0.004 f	22.15±0.89 b
$S_{150}T_0$	57.24±2.78 a	0.522±0.013 a	1.21±0.07 h	1.285±0.053 c	0.94±0.05 fg
$S_{150}T_1$	26.60±0.77 c	0.317±0.011 d	2.15±0.07 e	0.852±0.035 d	2.53±0.18 f
$S_{150}T_2$	23.62±1.56 c	0.287±0.011 d-f	1.82±0.04 f	1.465±0.060 b	1.24±0.07 fg
$S_{150}T_3$	44.58±1.92 b	0.460±0.021 b	1.39±0.04 g	1.329±0.054 c	1.05±0.06 fg
$S_{150}T_4$	19.26±0.57 c	0.212±0.010 g	2.83±0.09 d	0.646±0.034 e	4.40±0.36 e
$S_{150}T_5$	46.54±2.94 b	0.333±0.015 d	1.46±0.06 g	2.101±0.087 a	0.70±0.05 g
Tukey value/Significance	8.14**	0.051**	0.18**	0.119**	1.82**

Values are means ± standard error (n = 3). In each column, means followed by the same lowercase letter are not significantly different according to Tukey's honestly significant difference (HSD) test. Tukey value indicates the HSD value at the 0.01 level (**, $p < 0.01$). S_0: 0 mM NaCl, S_{150}: 150 mM NaCl. T_0: Control, T_1: PGPR, T_2: 0.50% WV, T_3: 1.00% WV, T_4: PGPR + 0.50% WV, T_5: PGPR + 1.00% WV (PGPR: Plant growth-promoting rhizobacteria, *Paenibacillus xylanilyticus* strain KF63C; WV: Wood vinegar, also known as pyroligneous acid).

Table S4. Effects of seed-inoculated PGPR and foliar-applied WV on photosynthetic pigment contents (Chlorophyll a, b, total chlorophyll, total carotenoids) and chlorophyll a/b ratio of broad bean (*Vicia faba* L.) under non-saline (0 mM NaCl) and saline (150 mM NaCl) conditions.

Treatments	Chlorophyll a (mg/g FW)	Chlorophyll b (mg/g FW)	Chlorophyll a/b Ratio	Total Chlorophyll (mg/g FW)	Total Carotenoids (mg/g FW)
S_0T_0	3.18±0.06 c	1.08±0.03 c	2.94±0.03 cd	4.26±0.09 c	0.80±0.02 d
S_0T_1	3.65±0.07 b	1.28±0.04 b	2.84±0.02 de	4.93±0.11 b	0.95±0.03 b
S_0T_2	3.26±0.06 c	1.12±0.03 c	2.90±0.02 c-e	4.38±0.08 c	0.83±0.02 cd
S_0T_3	2.87±0.04 d	0.90±0.02 de	3.19±0.03 b	3.77±0.06 d	0.66±0.02 ef
S_0T_4	3.88±0.07 a	1.41±0.04 a	2.75±0.02 e	5.29±0.11 a	1.03±0.05 a
S_0T_5	2.80±0.06 d	0.88±0.03 de	3.18±0.04 b	3.68±0.09 de	0.64±0.02 ef
$S_{150}T_0$	1.48±0.06 g	0.44±0.04 g	3.35±0.13 a	1.92±0.10 g	0.30±0.03 h
$S_{150}T_1$	2.58±0.04 e	0.85±0.03 e	3.04±0.06 bc	3.43±0.07 e	0.60±0.02 f
$S_{150}T_2$	2.79±0.05 d	0.96±0.02 d	2.92±0.02 cd	3.75±0.07 d	0.68±0.02 e
$S_{150}T_3$	1.79±0.06 f	0.54±0.03 f	3.34±0.05 a	2.33±0.08 f	0.38±0.02 g
$S_{150}T_4$	3.48±0.07 b	1.22±0.04 b	2.86±0.03 de	4.70±0.10 b	0.90±0.02 bc
$S_{150}T_5$	1.86±0.06 f	0.61±0.03 f	3.05±0.05 bc	2.47±0.09 f	0.41±0.02 g
Tukey value/Significance	0.17**	0.09**	0.15**	0.26**	0.07**

Values are means ± standard error (n = 3). In each column, means followed by the same lowercase letter are not significantly different according to Tukey's honestly significant difference (HSD) test. Tukey value indicates the HSD value at the 0.01 level (**, $p < 0.01$). S_0: 0 mM NaCl, S_{150}: 150 mM NaCl. T_0: Control, T_1: PGPR, T_2: 0.50% WV, T_3: 1.00% WV, T_4: PGPR + 0.50% WV, T_5: PGPR + 1.00% WV (PGPR: Plant growth-promoting rhizobacteria, *Paenibacillus xylanilyticus* strain KF63C; WV: Wood vinegar, also known as pyroligneous acid).
